# Celiac disease in paediatric patients in the United Arab Emirates: a single-center descriptive study

**DOI:** 10.3389/fped.2023.1197612

**Published:** 2023-07-18

**Authors:** Asma H. AlNababteh, Christos Tzivinikos, Saif Al-Shamsi, Romona Devi Govender, Rami H. Al-Rifai

**Affiliations:** ^1^ Institute of Public Health, College of Medicine and Health Sciences, United Arab Emirates University, Al-Ain, United Arab Emirates; ^2^ Department of Paediatric Gastroenterology, Al Jalila Children's Specialty Hospital, Dubai, United Arab Emirates; ^3^ Department of Internal Medicine, College of Medicine and Health Sciences, United Arab Emirates University, Al-Ain, United Arab Emirates; ^4^ Department of Family Medicine, College of Medicine and Health Sciences, United Arab Emirates University, Al-Ain, United Arab Emirates; ^5^ Zayed Center for Health Sciences, United Arab Emirates University, Al Ain, United Arab Emirates

**Keywords:** celiac disease, celiac, pediatric patients, gluten, United Arab Emirates

## Abstract

**Introduction:**

Celiac disease (CD) is an autoimmune disorder that is provoked by the consumption of gluten in genetically vulnerable individuals. CD affects individuals worldwide with an estimated prevalence of 1% and can manifest at any age. Growth retardation and anemia are common presentations in children with CD. The objective of this study is to estimate the prevalence of CD in multiple “at risk groups” and to characterize children with CD, presented to a tertiary hospital in Dubai, United Arab Emirates (UAE).

**Methods:**

The study reviewed medical charts of all patients <18 years who had received serologic testing for CD. The study was conducted at Al Jalila Children's Specialty Hospital in Dubai, UAE, from January 2018 to July 2021. Extracted information from medical records included sociodemographics, laboratory findings, clinical presentation, and any associated co-morbidities. The European Society of Paediatric Gastroenterology, Hepatology and Nutrition (ESPGHAN) criteria were used to identify patients with CD.

**Results:**

During the study period, 851 paediatric patients underwent serological screening for CD, out of which, 23 (2.7%) were confirmed with CD. Of the 23 patients diagnosed with CD, 43.5% had no gastrointestinal symptoms. Diabetes type 1 (30.4%) followed by iron deficiency anaemia (30%) and Hashimoto thyroiditis (9%) were the most commonly associated comorbidities. The prevalence of CD among paediatric patients with autoimmune thyroiditis (12.5%) was 1.92-times higher than that among paediatric patients with diabetes type 1 (6.5%).

**Conclusion:**

The results of this study show that almost three out of every 100 paediatric patients who were screened for CD were confirmed to have the condition. These findings highlight the importance of screening children who are at risk or present symptoms suggestive of CD, to ensure early diagnosis and appropriate management.

## Introduction

Celiac disease (CD) is becoming a growing public health issue with a rising prevalence worldwide (1). It is an autoimmune disorder that is provoked by the consumption of gluten in genetically vulnerable individuals. Gluten can be found in sources such as wheat, oats, rye, or barley (2). CD is characterized by small intestine enteropathy, which can lead to various intestinal and extraintestinal symptoms (3). In young children, the primary symptom of CD is diarrhea, which can lead to malabsorption and failure to thrive. Abdominal pain, vomiting, and constipation are more common in teenagers and older children with CD (4). Some children with CD may also present with extraintestinal manifestations such as anemia, arthritis, and neurological symptoms (5–7).

In recent decades, there has been a steady rise in the occurrence of CD, with an annual increase of 7.5% (1). The incidence and prevalence of CD showed regional and ethnic variations, as well as differences based on age and gender. According to a recent systematic review and meta-analysis, the estimated incidence of CD was 17.4 per 100,000 person-years for women and 7.8 per 100,000 person-years for men. The incidence of CD was higher among children, with a rate of 21.3 per 100,000 person-years, compared to adults, with a rate of 12.9 per 100,000 person-years (1).

To diagnose CD, suspected patients are initially screened for the presence of antibodies against tissue transglutaminase immunoglobulin A (tTG-IgA) and the total IgA in their serum (6). Studies have shown that screening for tTG IgA is the most reliable and cost-effective method for diagnosing CD, with a sensitivity and specificity of over 90% (8). If a patient has high levels of Anti tTG-IgA, a duodenal biopsy is typically recommended as the gold standard for confirming a diagnosis of CD (9). The European Society for Paediatric Gastroenterology, Hepatology and Nutrition (ESPGHAN), the National Institute for Health and Care Excellence (NICE), and the North American Society for Paediatric Gastroenterology Hepatology and Nutrition (NASPHGAN) have all recognized that certain “at-risk” groups have a higher prevalence of CD (10–12). Individuals with type 1 diabetes, unexplained anemia, Trisomy 21 (Down syndrome), delayed growth, and autoimmune thyroid disease are considered to be at higher risk for CD, and are recommended to be screened for the condition (10–12). ESPGHAN has established diagnostic criteria for CD in children, with a shift towards non-invasive and more accurate serological testing. The updated ESPGHAN guidelines, released in 2020, now allow for serological diagnosis of CD in children who meet certain clinical, genetic, and serologic criteria, without the need for biopsy confirmation (11). In patients with CD symptoms or without symptoms, a “no-biopsy” clinical approach may be used if the patient has a tTG-IgA serum level that is ten times the Upper Normal Level (UNL), along with a positive EMA in another blood sample.

Currently, there is a lack of population-based or clinic-based epidemiological data on the prevalence of CD among children in the United Arab Emirates (UAE). To address this, the objective of this study, conducted at Al Jalilah Children's Speciality Hospital (AJCH) in Dubai, is to determine the prevalence of CD among “at-risk” paediatric patients who were screened for the CD, and to examine the clinical presentations of patients who were diagnosed with CD.

## Methods

### Study setting

This study involved a chart review of all patients who underwent screening for CD at Al Jalilah Children's Speciality Hospital (AJCH) between January 1, 2018 and June 30, 2021. The hospital is located in the United Arab Emirates and is dedicated solely to treating children and adolescents up to 18 years old. All patients who were screened for CD either due to gastrointestinal symptoms or being in a high-risk group for developing the condition were included in the study. To document diseases in patients' electronic files, AJCH follows the International Classification of Diseases (ICD) codes, and the Current Procedural Terminology (CPT) codes are used to report the procedures and services provided to patients.

### Study population

All paediatric patients who were screened for CD at AJCH for various reasons were identified using the procedural codes 82784 and 83516, as well as the ICD code K90.0. Patients with a prior history of CD, those who had their screening performed at another hospital, or those who were on a gluten-free diet at the time of testing were excluded from the study. Sociodemographic characteristics, presenting symptoms, associated comorbidities, and laboratory findings were extracted from the electronic medical charts. The extracted data included age at diagnosis, gender, nationality, BMI-for-age percentile at the time of CD diagnosis, gastrointestinal symptoms, and comorbidities. Laboratory findings included total IgA, Anti-tTG IgA, EMA IgA, haemoglobin levels, ferritin levels, Vitamin D levels, and duodenal biopsy reports.

As per the Centre for Diseases Control and Prevention (CDC), BMI-for-age percentile was calculated using CDC online calculator to categorize the weight status of children and teens (13). Patients who lie below the 5th percentile on the growth chart were considered underweight, patients who were between the 5th and the 85th percentile on the chart were considered to have a healthy weight, patients who are between the 85th and the 95th were considered overweight and patients who have equal or more than 95th percentile were considered as obese.

### Serological investigation for CD

The AJCH follows the criteria set by the ESPGHAN to diagnose CD. The ESPGHAN 2012 criteria were initially utilized until the ESPGHAN 2020 criteria were introduced and subsequently adopted. Patients who are in any of the “at risk” groups of having CD (presented with gastrointestinal symptoms and/or having associated comorbidities) are tested for Anti-tTG IgA biomarkers and the total IgA. At AJCH, CD testing is performed by using a commercial kit “Chorus DIESSE Diagnostica Senese” based on the enzyme-linked immunosorbent assay (ELISA) approach (14). According to the manufacture, the sensitivity and the specificity of this testing kit are 97.1% and 100%, respectively, with no restrictions on the age. Following the manufacturer's instructions, 12 U/ml is the upper normal limit (UNL) for the Anti tTG IgA value. Patients with an anti-tTG IgA level of above 12 U/ml were considered positive. All patients who had values above 120 U/ml (>10× UNL) were called to test for anti endomysial antibodies (EMA). The EMA test was performed using a commercially available ELISA kit supplied by Euroimmun, Germany (15). Should the test yield a positive result then the diagnosis of CD is confirmed without the need for a duodenal biopsy as per the ESPGHAN criteria.

### Confirmation of CD diagnosis

Patients with an anti-tTG IgA level between 12 and 120 U/ml were called and offered an endoscopy and a duodenal biopsy was collected to evaluate histopathological changes in their duodenal mucosa. From each patient who have underwent endoscopic examination, four biopsies were collected from the duodenal mucosa with at least one from the duodenal bulb. The histopathologic examination was performed by an experienced pathologist and a Marsh score was assigned according to the severity of duodenal mucosal damage as follows; Marsh score 0: normal duodenal biopsy, Marsh score 1: mild damage that included intraepithelial lymphocytosis, Marsh score 2: lymphocytosis with crypt hyperplasia, Marsh score 3A: partial villous atrophy, Marsh score 3B: subtotal villous atrophy, and Marsh score 3C: total villous atrophy (16). Patients with a Marsh score of 2 or 3 were documented with a confirmed diagnosis of CD.

## Statistical analysis

The overall proportion of pediatric patients with serological-based and biopsy-based diagnosis of CD was quantified. Categorical characteristics of patients confirmed with CD presented as frequencies and percentages while continuous characteristics as means and standard deviations (SDs).

Data were analyzed using SPSS Statistics v26.0 software (IBM, Armonk, NY, USA).

## Results

### Scope of medical charts reviewing

**Figure 1:**
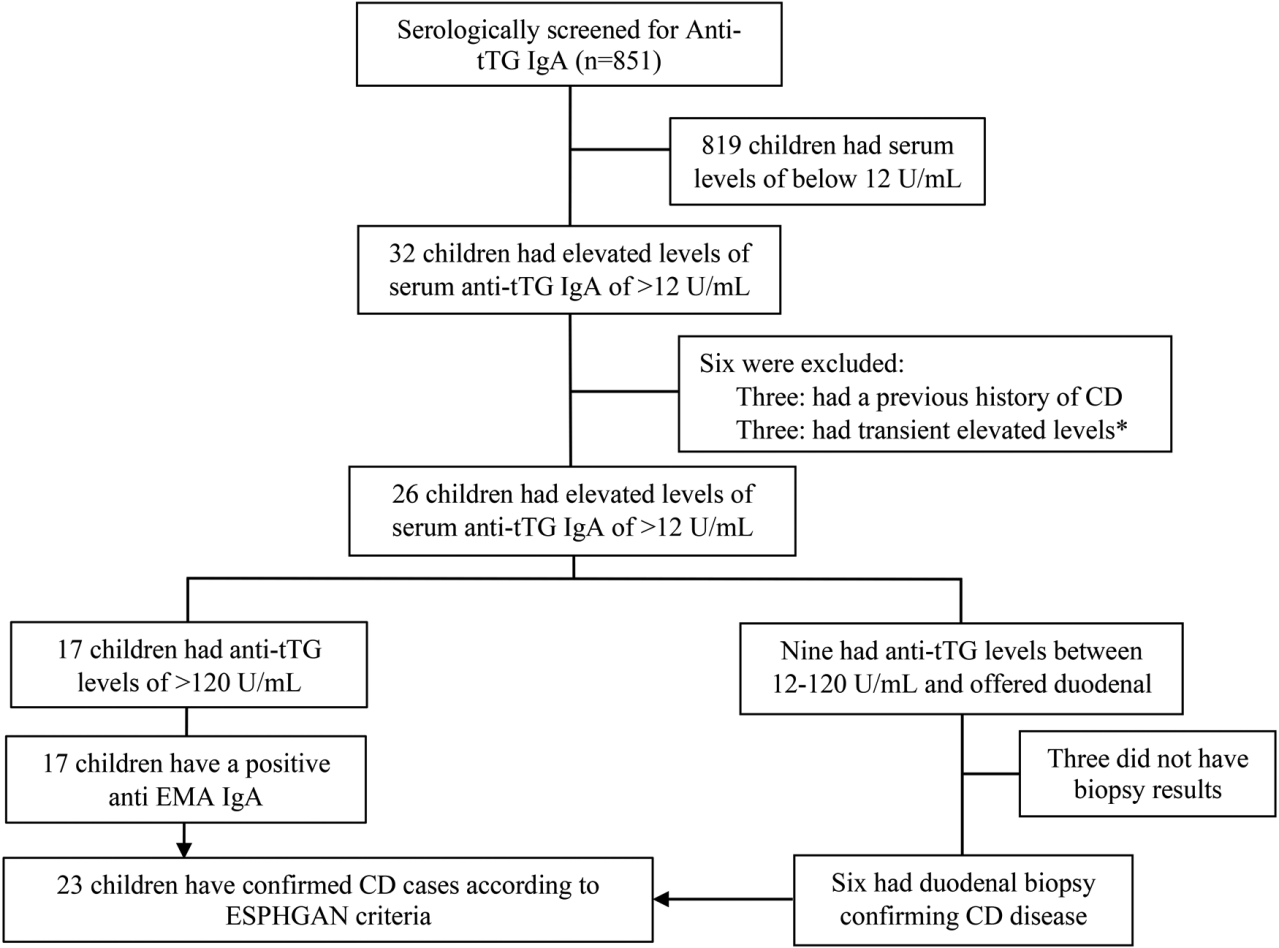
Flowchart of study population screened for CD at Al Jalilah children specialty hospital. * Excluded as this elevation was transient upon diagnosing the patient with Type 1 diabetes.

Over the course of the study period, a total of 851 medical records for paediatric patients who underwent serological screening for CD at AJCH were examined. Out of the screened patients, 32 individuals had an elevated (>12 U/ml) Anti-tTG IgA antibody levels. After excluding six of these patients, a total of 26 individuals (equivalent to 3.1% of the 845 patients) were identified as having CD. Of the 26 patients with CD, 17 had >120 U/ml and nine patients had 12–120 U/ml. According to the ESPGHAN guidelines, the 17 children with Anti-tTG IgA levels >120 U/ml were diagnosed with CD without the need for duodenal biopsy. Of the nine patients with Anti-tTG IgA antibodies level of 12–120 U/ml, parents of three children declined to have their children undergo duodenal biopsy, while six patients proceeded with the procedure; all of them had histopathological changes indicative to CD (four patients had Marsh grading of 3B and two patients had a Marsh grading of 3C). Overall, 23 (2.7%) of the 845 screened patients received a confirmed diagnosis of CD (Figure 1).

### Characteristics of paediatric patients confirmed with CD

On average, the 23 patients with CD were 6.7 (±3.3 SD) years old, with a majority of them being females (61%) and of Emirati nationality (69%). Out of the 23 paediatric patients who were diagnosed with CD, 22% were found to be underweight, with a BMI that fell below the 5th percentile for their age. 60% of the diagnosed patients had a healthy BMI, while 18% were classified as overweight or obese, with a BMI that was higher than the 85th percentile for their age. More than half (57.0%) of the paediatric patients were presented with gastrointestinal symptoms. The most prevalent comorbidity among patients with CD was Type 1 Diabetes, with 30.4% of patients exhibiting this condition. Table 1 shows that two (9.0%) patients diagnosed with CD also had autoimmune thyroiditis, and one patient had Down's syndrome (4.0%). Additionally, one patient presented with the triad of CD, Type 1 Diabetes, and autoimmune thyroiditis. In patients with CD, 30% of individuals had low haemoglobin levels for their age, with a mean value of 11.85 gm/dl. Among 18 patients with CD, four individuals (22%) had a low ferritin level. Vitamin D levels were assessed in 13 children, and 77% of them (10 individuals) had a Vitamin D level that was below 30 ng/ml (Table 1).

**Table 1:** Socio-demographic characteristics, co-morbidities, and laboratory findings of the included 23 CD patients who were confirmed based on ESPHGAN criteria.

	Patients with confirmed CD *n* = 23 (valid %)
**Sociodemographics**	
Age (mean ± SD), years	6.74 ± 3.3
**Gender**	
Males	9 (39.1)
Females	14 (60.9)
**Nationality**	
Emirati	16 (69.6)
Non-Emirati	7 (30.4)
**Children BMI for age**	
Underweight (less than 5th percentile)	5 (21.7)
Healthy weight (5^th^–85th percentile)	14 (60.9)
Overweight (85th–95th percentile)	2 (8.7)
Obese (>95th percentile)	2 (8.7)
**Gastrointestinal Symptoms**	
Yes	13 (56.5)
No	10 (43.5)
**Comorbidities**	
Diabetes type 1	7 (30.4)
Autoimmune thyroiditis	2 (8.7)
Down's Syndrome	1 (4.3)
**Laboratory findings**	
Anti-ttG IgA	
12–120 U/ml	6 (26.1)
>120 U/ml	17 (73.9)
Haemoglobin levels (mean ± SD g/dl)	11.85 ± 1.2
Anaemic^a^	7 (30.4)
Non anaemic	16 (69.6)
Ferritin (mean ± SD ng/ml)	29.6 ± 21.5
Low^b^	4 (22.2)
Normal	14 (77.8)
Vit D (mean ± SD ng/ml)	24.6 ± 6.8
Low^c^	10 (76.9)
Normal	3 (23.1)

^a^ Normal ranges for hemoglobin levels according to children age are available in Appendix 1 (Supplementary Material).

^b^ According to AJCH lab, levels below 5 ng/ml are considered low.

^c^ According to AJCH lab, levels below 30 ng/ml are considered low.

Table 2 displays data on children who were screened for CD as part of at-risk groups, rather than being assessed due to gastrointestinal symptoms. The highest prevalence of CD was observed among children diagnosed with autoimmune thyroiditis, with a prevalence of 12.5%. Following this, children with unexplained anemia had a prevalence rate of 7.1%, while those with Type 1 Diabetes, Down syndrome, and growth retardation had prevalence rates of 6.5%, 4.5%, and 2.2%, respectively (Table 2).

**Table 2:** Prevalence of CD among some “At-risk” groups screened at AJCH.

“At-risk” Group	Number (total no. screened)	CD confirmed cases (valid %)
Growth Retardation	133	3 (2.2)
Diabetes Type 1	107	7 (6.5)
Unexplained anaemia	28	2 (7.1)
Down Syndrome	22	1 (4.5)
Autoimmune Thyroiditis	16	2 (12.5)

## Discussion

In a major paediatric hospital in Dubai, this study explored the prevalence of CD among a group of children who were screened for the disease due to gastrointestinal symptoms or for being at-risk of developing the disease. According to the ESPGHAN criteria used in this study, the overall prevalence of CD among patients who were screened at AJCH during the study period was found to be 2.7%.

A recent systematic review and meta-analysis reported that the prevalence of undiagnosed CD in Europe ranged from 0.1% to 3.3%. The highest prevalence rates were reported in Spain and Sweden. Furthermore, this study showed a general increase in the annual trend of undiagnosed CD since 2000 (17). An increase in the prevalence of CD among children over the years was also observed in a twenty-year retrospective study based on the review of medical charts from two tertiary hospitals in the United States (18). The CD was previously thought to be primarily a disease of Western communities; however, recent reports have shown that it is also a common disorder in countries where gluten-containing meals are a staple food. For example, studies have reported CD prevalence rates of 5%–6% in Northern Africa and 0.4%–2.1% in the Middle East (19). Furthermore, parts of the Indian continent have also seen an increasing prevalence of CD, with changing clinical presentations.

Relating our results of CD prevalence in paediatric patients in the UAE to the previous reports we found that the observed prevalence is higher than many western countries. In the Gulf Cooperation Council (GCC) countries, there were more studies reporting CD prevalence and patients' characteristics in children than in adults. A study from Bahrain reported an overall prevalence of CD as 0.02%, however, this study might have underestimated the disease's prevalence by counting the cases from a single tertiary hospital records while considering the overall under 18 years Bahraini population as a denominator (20). A similar situation was seen in Kuwait, where an old study conducted in 1987 reported a CD prevalence rate of 1:3,000 births (21). However, at that time, there were no definite criteria for CD diagnosis, and the prevalence might have been underestimated. In Saudi Arabia, which is the largest country in the GCC, CD has been of interest to many researchers. A mass screening conducted in schools in Riyadh, the capital city of Saudi Arabia, revealed a CD prevalence rate of 1.5% (22). Another Saudi study conducted at the Eastern region of the Saudi Arabia reported a CD seroprevalence of 3% (23). These reports add to the high prevalence reported in some adults' studies (24, 25). The higher prevalence observed in our study compared to other studies in GCC countries could be attributed to the difference in those studies design, while our study included patients who were suspected of having CD in AJCH hospital, the other studies mentioned above were population-based done after actual general population screening. Additionally, there are several factors that contribute to the differences in CD prevalence between countries, including differences in genetics, environmental factors, and clinical practices. Improved recognition, screening, and diagnosis of CD can also lead to increased prevalence rates as more cases are identified. Additionally, increased awareness and understanding of CD among healthcare professionals and the general public can lead to more people seeking evaluation and diagnosis for the disease.

Similar to other epidemiological studies (24, 26, 27), the majority (60%) of the children diagnosed with CD were females. The preponderance of females having CD is most likely explained by the pathogenesis of autoimmunity as the autoimmune frequency has a female to male ratio of 2.7:1 (28). The association between HLA genes and a number of autoimmune disease (including CD) does exist (29) and that association shows a gender bias toward females in many studies (30, 31).

As discussed earlier, CD can occur at any age (32). The mean age in this study was 6.7 years which was similar to what have been previously reported (33, 34). According to the literature there are two distinct presentations of CD in infants and children, one is classical and occurs right after the introduction of gluten (age 6–24 months) characterized by chronic diarrhoea and impaired growth and the other is the non-classical and occurs between the age of (5–7 years) with symptoms of abdominal pain and short stature (35). However, among our CD patients the youngest age was 2.5 years, that might be contributed to the fact that the initial testing was done using tTG-IgA biomarker which previously reported to have a lower sensitivity in children who are less than two years old (36) Among the 23 children diagnosed with CD in our study, 22% were underweight according to their BMI for age value. While a large population based study have shown that children with CD are usually shorter and weigh less compared to their peers, that study of 12,632 couldn't provide evidence of the association between being underweight and the risk of developing CD (37). Although it is well established that children with CD are more likely to be underweight but as overweight and obesity among children continues to rise, the results around BMI for age are contradictory.

The associated risk of autoimmune diseases with CD is 3–10 times higher when compared to the general population (2). Thus, international guidelines advocate screening for CD in patients with autoimmune disorders such as type 1 DM, autoimmune thyroiditis, growth delay, unexplained anaemia and Down's syndrome as these are more prevalent in children with CD (9, 11, 12). Based on the prevalence of diabetes type 1 in the general population estimated to be 0.3% (38), the noteworthy finding of a high prevalence of 30% for type 1 diabetes among the patients confirmed with CD indicates the importance of screening for other autoimmune diseases in individuals with CD. Similarly, it emphasizes the need to screen for CD in patients diagnosed with any autoimmune disease.

On the other hand, our study estimated the prevalence of CD among multiple “at-risk” categories. The high prevalence of CD among diabetic patients supports the evidence from previous studies where the detection of CD was 6 times higher in type 1 diabetes patients than the general population (39–42). It is worth noting that in our study, all seven patients who were diagnosed with both diabetes and CD did not show any symptoms of CD, and their CD would have gone undiagnosed if they were not screened based on the international screening guidelines.

Another autoimmune disease that was reported in two patients was the autoimmune thyroid disease. It is well known that patients with CD are at increased risk of developing thyroid disease and that was evident in a large population study conducted in Italy where individuals with CD had 4.64 increased risk of developing hypothyroidism (43). On the other hand, the prevalence of CD was also higher in children with autoimmune thyroid disease than the general population. CD pooled prevalence of 6,024 patients with autoimmune thyroid disease was reported as 2.6% in a meta-analysis published in 2015 (44). The above mentioned suggest that screening for CD in patients who were diagnosed with other autoimmune diseases is mandatory and might save the children from a severe form of CD (45).

In the present study, a main reason to screen paediatric patients for CD was the delay in growth. Of the 133 who had growth retardation, three were diagnosed with CD. The main reason for children with CD to be shorter and with less weight than their peers is the gastrointestinal malabsorption which leads to nutritional deficiency (46, 47). Moreover, some of CD patients had a growth hormone deficiency which might cause a marked delay in growth. That coexistence of CD and growth hormone deficiency has been reported in a recent prospective multicenter Saudi study where children's growth was improved after growth hormone replacement therapy (48). Unfortunately, in our study there are no further details on the growth hormone levels among the patients and therefor can't relate both factors to each other.

Anaemia was identified according to the children's age-based haemoglobin levels. Low haemoglobin levels were evident in 30% of CD patients, in addition to that, unexplained anaemia was the sole reason for screening in 28 of the children screened. The association between CD in children and anaemia is well observable in previous studies (49, 50). In our patients, as a manifestation of micronutrients absorption impairment, four patients (22%) of the children had low levels of haemoglobin, serum ferritin and Vit D and that was expressed by severe damage in their intestinal mucosa with near total villous atrophy of the intestinal villi.

Multiple syndromes have also been associated with CD. The association between CD and Down's syndrome has been studied in a nationwide Swedish case-control study where researchers found a significantly increased prevalence of Down's syndrome among the CD patients as compared to 53,887 CD free controls (51). Due to the limited sample size in our study, a comprehensive investigation into the genuine association between Down's syndrome and CD was unattainable. This was due to the fact that out of the 22 pediatric patients with Down's syndrome, only one of them was diagnosed with CD.

There are important limitations to consider when interpreting the findings of the study. It is important to note that future studies with larger sample sizes and a comparator group from the general population may provide a more comprehensive understanding of CD prevalence and associated factors in the UAE population. Hence, findings in this study should not be generalized to whole population in the UAE. Additionally, it would be beneficial for future studies to collect additional data on factors such as growth hormone levels to better understand the relationship between CD and growth delay. Additionally, future studies may consider exploring the cost-effectiveness of such screening measures for CD among “at-risk” populations.

Nevertheless, this is the first study to provide a foundation for further studies to be conducted in the UAE to gain a better understanding of the prevalence and characteristics of CD in children, associated comorbidities, and the long-term outcomes of these children. The identification of patients with CD was based on serological investigation of Anti-tTG IgA and confirmation histopathologically. Reporting on the prevalence of CD among “at-risk” groups supports prioritizing specific patients for CD screening.

## Conclusion

Three out of every 100 paediatric patients who were screened for CD were confirmed to have the condition. Early diagnosis and management of CD can lead to improved health outcomes and prevent long-term complications. Therefore, screening children who have symptoms or belong to at-risk groups can be a cost-effective way to identify and manage CD in a timely manner.

## Data Availability

The raw data supporting the conclusions of this article will be made available by the authors, upon justifiable reasons and after receiving official approvals. Requests to access the datasets should be directed to rrifai@uaeu.ac.ae.

## References

[1] King JA Jeong J Underwood FE Quan J Panaccione N Windsor JW Incidence of celiac disease is increasing over time: a systematic review and meta-analysis. Am J Gastroenterol. (2020) 115(4):507–25. 10.14309/ajg.0000000000000523 32022718

[2] Sahin Y. Celiac disease in children: a review of the literature. World J Clin Pediatr. (2021) 10(4):53. 10.5409/wjcp.v10.i4.53 34316439 PMC8290992

[3] Lebwohl B Rubio-Tapia A. Epidemiology, presentation, and diagnosis of celiac disease. Gastroenterology. (2021) 160(1):63–75. 10.1053/j.gastro.2020.06.098 32950520

[4] Rampertab SD Pooran N Brar P Singh P Green PH. Trends in the presentation of celiac disease. Am J Med. (2006) 119(4):355.e9–14. 10.1016/j.amjmed.2005.08.044 16564784

[5] Ghiselli A Bizzarri B Gaiani F Semeraro F Iuliano S Di Mario F Growth changes after gluteen free diet in pediatric celiac patients: a literature-review. Acta Biomed. (2018) 89(9-s):5–10. 10.23750/abm.v89i9-S.7871 30561389 PMC6502184

[6] Barker JM Liu E. Celiac disease: pathophysiology, clinical manifestations, and associated autoimmune conditions. Adv Pediatr. (2008) 55:349–65. 10.1016/j.yapd.2008.07.001 19048738 PMC2775561

[7] Trovato CM Raucci U Valitutti F Montuori M Villa MP Cucchiara S Neuropsychiatric manifestations in celiac disease. Epilepsy Behav. (2019) 99:106393. 10.1016/j.yebeh.2019.06.036 31479999

[8] Kaswala DH Veeraraghavan G Kelly CP Leffler DA. Celiac disease: diagnostic standards and dilemmas. Diseases. (2015) 3(2):86–101. 10.3390/diseases3020086 28943611 PMC5548238

[9] Hill ID Fasano A Guandalini S Hoffenberg E Levy J Reilly N NASPGHAN clinical report on the diagnosis and treatment of gluten-related disorders. J Pediatr Gastroenterol Nutr. (2016) 63(1):156–65. 10.1097/MPG.0000000000001216 27035374

[10] Hill ID Dirks MH Liptak GS Colletti RB Fasano A Guandalini S Guideline for the diagnosis and treatment of celiac disease in children: recommendations of the North American society for pediatric gastroenterology, hepatology and nutrition. J Pediatr Gastroenterol Nutr. (2005) 40(1):1–19. 10.1097/00005176-200501000-00001 15625418

[11] Husby S Koletzko S Korponay-Szabó I Kurppa K Mearin ML Ribes-Koninckx C European society paediatric gastroenterology, hepatology and nutrition guidelines for diagnosing coeliac disease 2020. J Pediatr Gastroenterol Nutr. (2020) 70(1):141–56. 10.1097/MPG.0000000000002497 31568151

[12] Downey L Houten R Murch S Longson D. Recognition, assessment, and management of coeliac disease: summary of updated NICE guidance. BMJ. (2015):351:h4513. 10.1136/bmj.h4513 26333593

[13] Kuczmarski RJ Ogden CL Grummer-Strawn LM Flegal KM Guo SS Wei R CDC Growth charts: United States advance data from vital and health statistics, no. 314. Hyattsville, MD: National Center for Health Statistics (2000). 11183293

[14] Diesse. CHORUS tTg-A. For the semiquantitative determination of IgA antibodies anti-tissue Transglutaminase. (2015).

[15] Gosink J. Diagnosis of Coeliac Disease: Moving beyond biopsy. Available at: https://www.euroimmun.com/fileadmin/user_upload/News/Professional-articles/HV_3011_L_UK_C.pdf

[16] Oberhuber G. Histopathology of celiac disease. Biomed Pharmacother. (2000) 54(7):368–72. 10.1016/S0753-3322(01)80003-2 10989975

[17] Roberts SE Morrison-Rees S Thapar N Benninga MA Borrelli O Broekaert I Systematic review and meta-analysis: the incidence and prevalence of paediatric coeliac disease across Europe. Aliment Pharmacol Ther. (2021) 54(2):109–28. 10.1111/apt.16337 34115894

[18] Almallouhi E King KS Patel B Wi C Juhn YJ Murray JA Increasing incidence and altered presentation in a population-based study of pediatric celiac disease in North America. J Pediatr Gastroenterol Nutr. (2017) 65(4):432–7. 10.1097/MPG.0000000000001532 28151767 PMC5538895

[19] Barada K Bitar A Mokadem MA Hashash JG Green P. Celiac disease in Middle Eastern and North African countries: a new burden? World J Gastroenterol. (2010) 16(12):1449–57. 10.3748/wjg.v16.i12.1449 20333784 PMC2846249

[20] Isa HM Farid E Makhlooq JJ Mohamed AM Al-Arayedh JG Alahmed FA Celiac disease in children: increasing prevalence and changing clinical presentations. Clin Exp Pediatr. (2021) 64(6):301–9. 10.3345/cep.2020.00304 33091973 PMC8181022

[21] Khuffash FA Barakat MH Shaltout AA Farwana SS Adnani MS Tungekar MF. Coeliac disease among children in Kuwait: difficulties in diagnosis and management. Gut. (1987) 28(12):1595–9. 10.1136/gut.28.12.1595 3428686 PMC1433935

[22] Al-Hussaini A Troncone R Khormi M AlTuraiki M Alkhamis W Alrajhi M Mass screening for celiac disease among school-aged children: toward exploring celiac iceberg in Saudi Arabia. J Pediatr Gastroenterol Nutr. (2017) 65(6):646–51. 10.1097/MPG.0000000000001681 28753180

[23] Al Hatlani MM. Prevalence of celiac disease among symptom-free children from the eastern province of Saudi Arabia. Saudi J Gastroenterol. (2015) 21(6):367–71. 10.4103/1319-3767.170952 26655131 PMC4707804

[24] Aljebreen AM Almadi MA Alhammad A Al Faleh FZ. Seroprevalence of celiac disease among healthy adolescents in Saudi Arabia. World J Gastroenterol. (2013) 19(15):2374–8. 10.3748/wjg.v19.i15.2374 23613632 PMC3631990

[25] Khayyat YM. Serologic markers of gluten sensitivity in a healthy population from the western region of Saudi Arabia. Saudi J Gastroenterol. (2012) 18(1):23–5. 10.4103/1319-3767.91733 22249088 PMC3271689

[26] Singh P Arora A Strand TA Leffler DA Catassi C Green PH Global prevalence of celiac disease: systematic review and meta-analysis. Clin Gastroenterol Hepatol. (2018) 16(6):823–36.e2. 10.1016/j.cgh.2017.06.037 29551598

[27] Ashtari S Najafimehr H Pourhoseingholi MA Rostami K Asadzadeh-Aghdaei H Rostami-Nejad M Prevalence of celiac disease in low and high risk population in Asia-pacific region: a systematic review and meta-analysis. Sci Rep. (2021) 11(1):2383. 10.1038/s41598-021-82023-8 33504878 PMC7841177

[28] Jacobson DL Gange SJ Rose NR Graham NM. Epidemiology and estimated population burden of selected autoimmune diseases in the United States. Clin Immunol Immunopathol. (1997) 84(3):223–43. 10.1006/clin.1997.4412 9281381

[29] Ngo ST Steyn FJ McCombe PA. Gender differences in autoimmune disease. Front Neuroendocrinol. (2014) 35(3):347–69. 10.1016/j.yfrne.2014.04.004 24793874

[30] Avidan N Le Panse R Berrih-Aknin S Miller A. Genetic basis of myasthenia gravis–a comprehensive review. J Autoimmun. (2014) 52:146–53. 10.1016/j.jaut.2013.12.001 24361103

[31] Czaja AJ Donaldson PT. Gender effects and synergisms with histocompatibility leukocyte antigens in type 1 autoimmune hepatitis. Am J Gastroenterol. (2002) 97(8):2051–7. 10.1111/j.1572-0241.2002.05921.x 12190176

[32] Caio G Volta U Sapone A Leffler DA De Giorgio R Catassi C Celiac disease: a comprehensive current review. BMC Med. (2019) 17(1):142. 10.1186/s12916-019-1380-z 31331324 PMC6647104

[33] Krauthammer A Guz-Mark A Zevit N Marderfeld L Waisbourd-Zinman O Silbermintz A Age-dependent trends in the celiac disease: a tertiary center experience. J Pediatr Gastroenterol Nutr. (2021) 72(6):894–9. 10.1097/MPG.0000000000003130 33908739

[34] Saeed A Assiri A Assiri H Ullah A Rashid M. Celiac disease in Saudi children. Evaluation of clinical features and diagnosis. Saudi Med J. (2017) 38(9):895–9. 10.15537/smj.2017.9.20808 28889146 PMC5654022

[35] Fasano A Catassi C. Coeliac disease in children. Best Pract Res Clin Gastroenterol. (2005) 19(3):467–78. 10.1016/j.bpg.2005.01.008 15925850

[36] Catassi GN Pulvirenti A Monachesi C Catassi C Lionetti E. Diagnostic accuracy of IgA anti-transglutaminase and IgG anti-deamidated gliadin for diagnosis of celiac disease in children under two years of age: a systematic review and meta-analysis. Nutrients. (2021) 14(1):7. 10.3390/nu14010007 35010880 PMC8746847

[37] van der Pals M Myléus A Norström F Hammarroth S Högberg L Rosén A Body mass index is not a reliable tool in predicting celiac disease in children. BMC Pediatr. (2014) 14(1):165. 10.1186/1471-2431-14-165 24981433 PMC4094403

[38] Los E Wilt AS. Diabetes Mellitus type 1 in children. StatPearls. Treasure Island (FL): StatPearls Publishing Copyright © 2022, StatPearls Publishing LLC. (2022).

[39] Elfström P Sundström J Ludvigsson JF. Systematic review with meta-analysis: associations between coeliac disease and type 1 diabetes. Aliment Pharmacol Ther. (2014) 40(10):1123–32. 10.1111/apt.12973 25270960

[40] Taczanowska A Schwandt A Amed S Tóth-Heyn P Kanaka-Gantenbein C Volsky SK Celiac disease in children with type 1 diabetes varies around the world: an international, cross-sectional study of 57 375 patients from the SWEET registry. J Diabetes. (2021) 13(6):448–57. 10.1111/1753-0407.13126 33118261

[41] Reilly NR Green PHR. Epidemiology and clinical presentations of celiac disease. Semin Immunopathol. (2012) 34(4):473–8. 10.1007/s00281-012-0311-2 22526468

[42] Dehbozorgi M Honar N Ekramzadeh M Saki F. Clinical manifestations and associated disorders in children with celiac disease in southern Iran. BMC Pediatr. (2020) 20(1):256. 10.1186/s12887-020-02162-1 32460713 PMC7251905

[43] Canova C Pitter G Ludvigsson JF Romor P Zanier L Zanotti R Celiac disease and risk of autoimmune disorders: a population-based matched birth cohort study. J Pediatr. (2016) 174:146–52.e1. 10.1016/j.jpeds.2016.02.058 27021409

[44] Roy A Laszkowska M Sundström J Lebwohl B Green PH Kämpe O Prevalence of celiac disease in patients with autoimmune thyroid disease: a meta-analysis. Thyroid. (2016) 26(7):880–90. 10.1089/thy.2016.0108 27256300

[45] Singh P Singh AD Ahuja V Makharia GK. Who to screen and how to screen for celiac disease. World J Gastroenterol. (2022) 28(32):4493–507. 10.3748/wjg.v28.i32.4493 36157923 PMC9476868

[46] Nemet D Raz A Zifman E Morag H Eliakim A. Short stature, celiac disease and growth hormone deficiency. J Pediatr Endocrinol Metab. (2009) 22(10):979–83. 10.1515/JPEM.2009.22.10.979 20020588

[47] Friedman A. Micronutrient deficiencies in pediatric celiac disease. ICAN Infant Child Adolescent Nutr. (2012) 4(3):156–67. 10.1177/1941406412440594

[48] Qutub LM Saadah OI. Prevalence of combined growth hormone deficiency and celiac disease among Saudi Arabian children with short stature: a tertiary care center experience. Chin Med J. (2020) 6:729–31. 10.1097/CM9.0000000000000715 PMC7190224 32197032

[49] Leffler DA Green PH Fasano A. Extraintestinal manifestations of coeliac disease. Nat Rev Gastroenterol Hepatol. (2015) 12(10):561–71. 10.1038/nrgastro.2015.131 26260366

[50] Pinto-Sánchez MI Bercik P Verdu EF Bai JC. Extraintestinal manifestations of celiac disease. Dig Dis. (2015) 33(2):147–54. 10.1159/000369541 25925916

[51] Mårild K Stephansson O Grahnquist L Cnattingius S Söderman G Ludvigsson JF. Down syndrome is associated with elevated risk of celiac disease: a nationwide case-control study. J Pediatr. (2013) 163(1):237–42. 10.1016/j.jpeds.2012.12.087 23399451

