# Supplementary material for: Celiac disease in paediatric patients in the United Arab Emirates: a single-center descriptive study
Source: Front Pediatr. 2023 Jul 18;11:1197612. doi: 10.3389/fped.2023.1197612 (PMC10391541; doi:10.3389/fped.2023.1197612)
Supplement: Supplementary file 1 [file Table1.docx]

|  | Age | | Gender | Lower Limit | Higher Limit | Unit |
| --- | --- | --- | --- | --- | --- | --- |
| Hemoglobin | 0 Minutes | 1 Days | Male | 14 | 22 | gm/dL |
| Hemoglobin | 1 Days | 3 Days | Male | 15 | 21 | gm/dL |
| Hemoglobin | 3 Days | 7 Days | Male | 13.5 | 21.5 | gm/dL |
| Hemoglobin | 7 Days | 14 Days | Male | 12.5 | 20.5 | gm/dL |
| Hemoglobin | 14 Days | 1 Months | Male | 12.2 | 18 | gm/dL |
| Hemoglobin | 1 Months | 2 Months | Male | 9.4 | 13 | gm/dL |
| Hemoglobin | 2 Months | 6 Months | Male | 11.1 | 14.1 | gm/dL |
| Hemoglobin | 6 Months | 1 Years | Male | 11.1 | 13.1 | gm/dL |
| Hemoglobin | 1 Years | 6 Years | Male | 11 | 14 | gm/dL |
| Hemoglobin | 6 Years | 12 Years | Male | 11.5 | 15.5 | gm/dL |
| Hemoglobin | 12 Years | 150 Years | Male | 13 | 17 | gm/dL |
| Hemoglobin | 0 Minutes | 1 Days | Female | 14 | 22 | gm/dL |
| Hemoglobin | 1 Days | 3 Days | Female | 15 | 21 | gm/dL |
| Hemoglobin | 3 Days | 7 Days | Female | 13.5 | 21.5 | gm/dL |
| Hemoglobin | 7 Days | 14 Days | Female | 12.5 | 20.5 | gm/dL |
| Hemoglobin | 14 Days | 1 Months | Female | 12.2 | 18 | gm/dL |
| Hemoglobin | 1 Months | 2 Months | Female | 9.4 | 13 | gm/dL |
| Hemoglobin | 2 Months | 6 Months | Female | 11.1 | 14.1 | gm/dL |
| Hemoglobin | 6 Months | 1 Years | Female | 11.1 | 13.1 | gm/dL |
| Hemoglobin | 1 Years | 6 Years | Female | 11 | 14 | gm/dL |
| Hemoglobin | 6 Years | 12 Years | Female | 11.5 | 15.5 | gm/dL |
| Hemoglobin | 12 Years | 150 Years | Female | 12 | 15 | gm/dL |
| Hemoglobin | 0 Minutes | 150 Years | Unknown | 13 | 17 | gm/dL |

Appendix 1: Normal ranges of hemoglobin levels.
